# Ground reaction force and electromyograms of lower limb muscles during fast walking

**DOI:** 10.3389/fspor.2022.1055302

**Published:** 2023-02-17

**Authors:** Akitoshi Makino, Keiichi Yamaguchi, Daichi Sumi, Masaru Ichikawa, Masumi Ohno, Akinori Nagano, Kazushige Goto

**Affiliations:** ^1^ Graduate School of Sport and Health Science, Ritsumeikan University, Shiga, Japan; ^2^ Research Fellow of Japan Society for the Promotion of Science, Tokyo, Japan; ^3^ Research Center for Urban Health and Sports, Osaka City University, Osaka, Japan; ^4^ Institute of Sport Science, ASICS Corporation, Hyogo, Japan; ^5^ Business Incubation Department, ASICS Corporation, Hyogo, Japan

**Keywords:** fast walking, running, ground reaction force, electromyogram, health promotion

## Abstract

**Background:**

Physically active status is an important contributor to individual health. Walking is regarded as commonly accepted exercise for exercise promotion. Particularly, interval fast walking (FW), consisting of alternating between fast and slow walking speeds, has gained popularity from practical viewpoints. Although previous studies have determined the short- and long-term effects of FW programs on endurance capacity and cardiovascular variables, factors affecting these outcomes have not been clarified. In addition to physiological variables, understanding of mechanical variables and muscle activity during FW would be a help to understand characteristics of FW. In the present study, we compared the ground reaction force (GRF) and lower limb muscle activity between fast walking (FW) and running at equivalent speeds.

**Method:**

Eight healthy men performed slow walking (45% of the maximum walking speed; SW, 3.9 ± 0.2 km/h), FW (85% of the maximum walking speed, 7.4 ± 0.4 km/h), and running at equivalent speeds (Run) for 4 min each. GRF and average muscle activity (aEMG) were evaluated during the contact, braking, and propulsive phases. Muscle activities were determined for seven lower limb muscles: gluteus maximus (GM), biceps femoris (BF), rectus femoris (RF), vastus lateralis (VL), gastrocnemius medialis (MG), soleus (SOL), and tibialis anterior (TA).

**Results:**

The anteroposterior GRF was greater in FW than in Run during the propulsive phase (p < 0.001), whereas the impact load (peak and average vertical GRF) was lower in FW than in Run (p < 0.001). In the braking phase, lower leg muscle aEMGs were higher during Run than during SW and FW (p < 0.001). However, in the propulsive phase, soleus muscle activity was greater during FW than during Run (p < 0.001). aEMG of tibialis anterior was higher during FW than during SW and Run in the contact phase (p < 0.001). No significant difference between FW and Run was observed for HR and RPE.

**Conclusion:**

These results suggest that the average muscle activities of lower limbs (e.g., gluteus maximus, rectus femoris, and soleus) during the contact phase were comparable between FW and running, however, the activity patterns of lower limb muscles differed between FW and running, even at equivalent speeds. During running, muscles were mainly activated in the braking phase related to impact. In contrast, during FW, soleus muscle activity during the propulsive phase was increased. Although cardiopulmonary response was not different between FW and running, exercise using FW might be useful for health promotion among individuals who cannot exercise at high-intensity.

## 1. Introduction

Daily physical activity is associated with improved cardiopulmonary function (1) and increased muscle mass and strength (2). Reduced physical activity leads to metabolic disturbances and related diseases, including insulin resistance (3), hyperglycemia (4), and atherosclerosis (5). Increased energy expenditure (EE) during exercise also reduces the risk of cardiovascular diseases (6). Therefore, strategies to increase exercise-induced EE are essential for health promotion.

Walking is widely accepted exercise modality among the adults (7, 8). Particularly, interval fast walking (FW), consisting of alternating between fast and slow walking speeds, has gained popularity from practical viewpoint. Cycling (pedaling) exercise is generally easy to perform but requires a stationary bike. Running does not require equipment but may pose risks of injury for those with orthopedic or other medical problems. On the other hand, it was reported that walking has a lower risk of injury than running (9). Previous studies have demonstrated improvements in maximal oxygen uptake, blood pressure, and heart rate after 5 months of FW (≥ 5 sets of FW at 70%–85% of peak aerobic capacity followed by slow walking at ≤ 40% of peak aerobic capacity; 3 min per set) (10, 11). Interval FW for 2 weeks improved insulin sensitivity, while reducing 24 h maximum glucose levels and mean amplitudes of glycemic excursion, in patients with type 2 diabetes (12).

Although previous studies have established the short- and long-term effects of FW programs on endurance capacity and cardiovascular variables, factors influencing these outcomes have not been investigated (10, 12). In our previous study, we demonstrated that EE and carbohydrate oxidation during walking were enhanced in a non-linear manner with increasing speed. It was notable that walking at speeds > 8.0 km/h caused greater EE and carbohydrate oxidation than running at an equivalent speed in young individuals (13). Moreover, previous studies reported that interval walking for 17 weeks or 5 months increased muscle strength of knee extensors and flexors muscles (14, 15),. Although Kubo et al. (16) reported that a walking exercise program for six months increased muscle strength of the knee flexors, no significant increase in muscle strength of the knee extensors was found.

In addition to physiological variables, understanding of mechanical variables and muscle activity during FW would be a help to clarify characteristics of FW. Walking speeds influence biomechanical variables such as joint kinematics, GRFs, joint moments of moments and powers, muscle activities, and spatiotemporal gait parameters (17). Previous studies compared GRFs (18) and muscle activity of the lower limbs (19) among different walking speeds. However, in these studies, no significant differences in GRFs were found between slow and normal speeds. Hence, comparisons of both GRFs and lower limbs of muscle activity between FW and running are currently required.

Therefore, the purpose of the present study was to compare the ground reaction force (GRF) and lower limb muscle activity between FW and running at equivalent speeds. We hypothesized that the GRF would be lower during FW than during running. Moreover, we hypothesized that lower limb muscle activity during FW would be mainly enhanced in the propulsive phase, whereas it would be activated in the braking phase while running.

## 2. Materials and methods

### 2.1. Participants

Eight men were recruited in the present study (mean ± standard deviation: age, 22 ± 1 y; height, 172.1 ± 1.7 cm; weight, 62.1 ± 7.0 kg); they received an overview of the experiment and possible risks (Table 1). None of them had any history of chronic diseases that could affect neuromuscular function, exercise, or daily physical activity. All participants were not involved in any training programs at the start of the study. Written informed consent was obtained from all participants. This study was approved by the ethics committee for Human Experiments at Ritsumeikan University (BKC-IRB-2020–047) and was conducted in accordance with the Declaration of Helsinki.

**Table 1:** Physical characteristics of the subjects and walking speed variables.

Age	22 ± 1 Year
Height	172.1 ± 1.7 cm
Weight	62.1 ± 7.0 kg
45% MWS (slow walk)	3.9 ± 0.2 km/h
85% MWS (Fast walk and run)	7.4 ± 0.4 km/h
MWS	8.8 ± 0.5 km/h

Values are means ± SD.

### 2.2. Experimental overview

Participants visited the laboratory twice throughout the experimental period. On the first visit, a familiarization session and determination of the maximal walking speed (MWS) were conducted. On the second visit, each participant performed the main experimental trials, consisting of slow walk trial, FW and Run trial. The anteroposterior and vertical GRF components, surface electromyography (EMG) of lower limb muscles were evaluated during each trial.

### 2.3. Exercise protocol

#### 2.3.1. MWS measurements

The participants began walking on a treadmill (Elevation series E95Ta; Life Fitness Corp., Franklin Park, IL, United States) at a speed of 4.0 km/h. The speed was progressively increased by 1.0 km/h at 1 min intervals until the participants could no longer match the speed; this speed was recorded as the MWS.

#### 2.3.2. Main experiment

**Figure 1:**
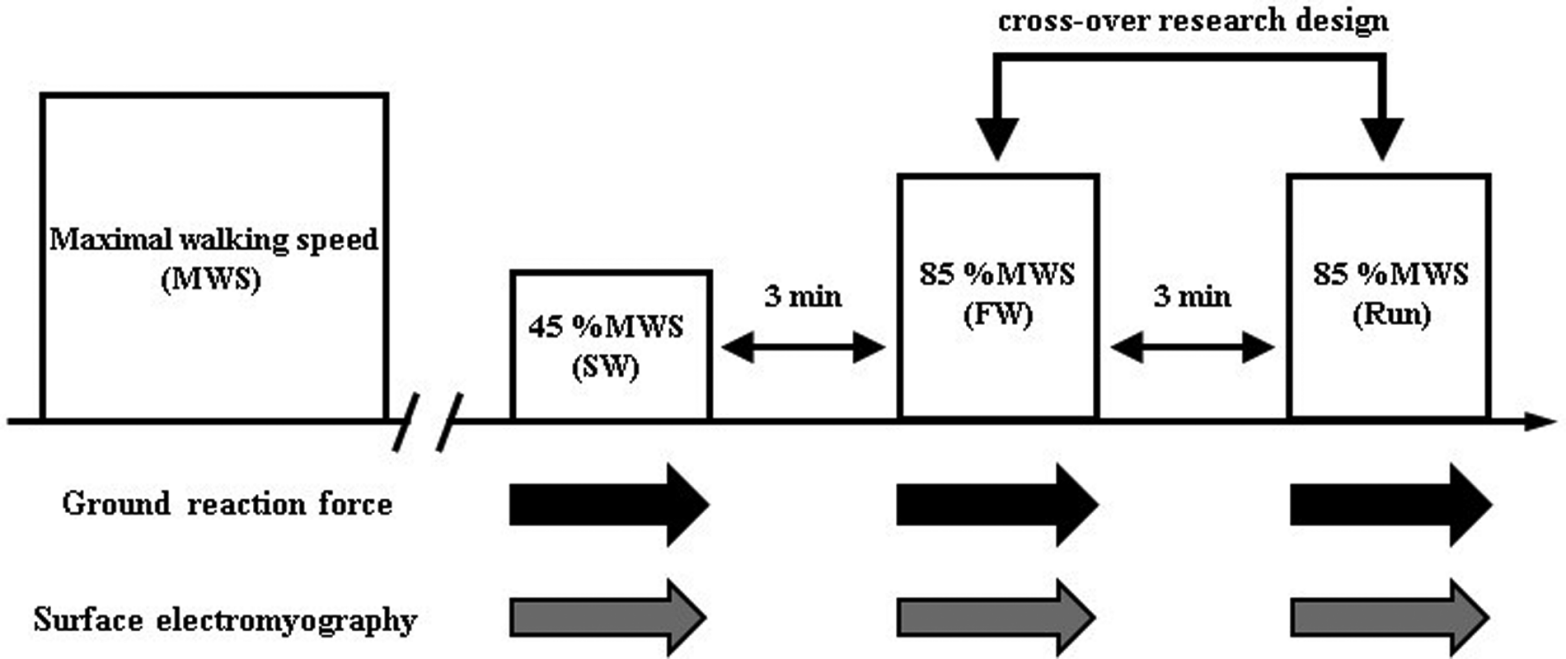
Protocol overview.

The participants walked for 4 min at 45% of MWS (slow walk) on a special treadmill with built-in force plates (HPT-2200D; Tec Gihan Co., Ltd., Kyoto, Japan); they then ran (Run) or walked at 85% of MWS (fast walk) for 4 min. The exercises were separated by 3-min rest periods. The order of running and FW was randomized. Based on our previous study which revealed significantly greater EE and carbohydrate oxidation in FW than in running (13), we selected 85% of MWS during fast walk phase. Heart rate (HR), rating of perceived exertion (RPE), GRF, and EMG were measured during the exercise (Figure 1).

#### 2.3.3. GRF and EMG measurements

**Figure 2:**
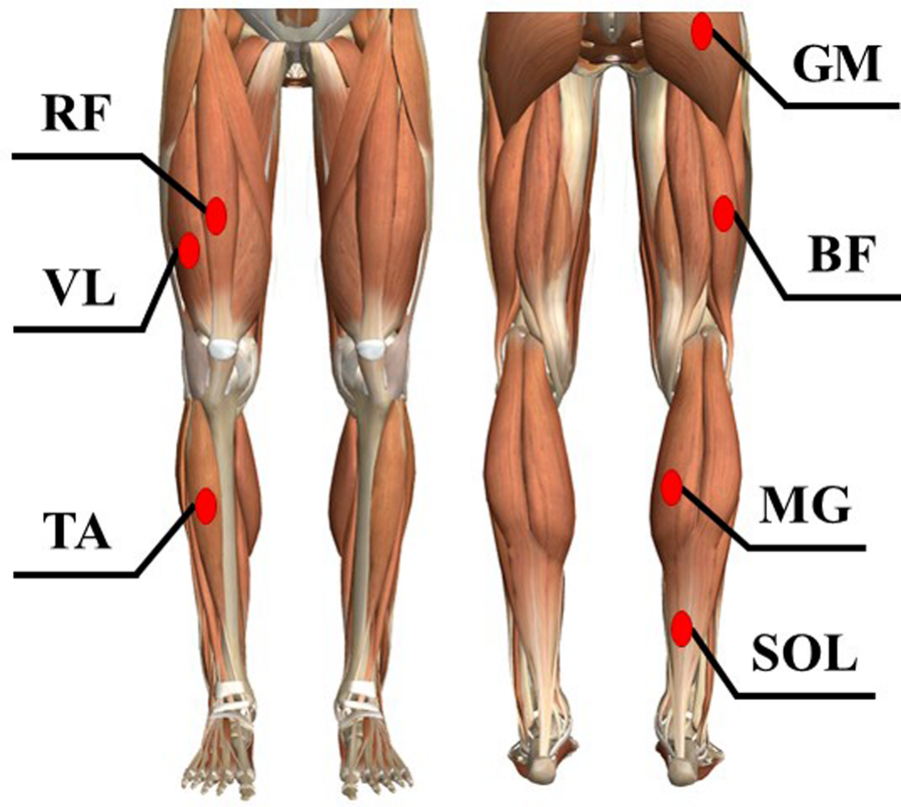
The measurement places of surface electromyography (EMG) of lower limb muscles. The EMGs were recorded and amplified from seven right lower limb muscles. GM; gluteus maximus, BF; biceps femoris, RF; rectus femoris, VL; vastus lateralis, MG; gastrocnemius medialis, SOL; soleus, TA; tibialis anterior.

A dual-belt treadmill with two force plates (HPT-2200D; Tec Gihan Co., Ltd.) was used for GRF measurements. Surface EMGs were recorded and amplified (SX230–1000, Biometrics Ltd., Ghent, United Kingdom) from seven right lower limb muscles: gluteus maximus (GM), biceps femoris (BF), rectus femoris (RF), vastus lateralis (VL), gastrocnemius medialis (MG), soleus (SOL), and tibialis anterior (TA). EMG electrode placement was based on the guidelines for non-invasive surface EMG assessment of muscles (20) (Figure 2). GRFs and EMGs were recorded at a sampling frequency of 1 kHz using a data acquisition and analysis system (LabChart; ADInstruments, Sydney, Australia) with a 16-bit analog-to-digital converter (PowerLab/16SP; ADInstruments).

#### 2.3.4. HR and RPE

HR was measured continuously (every 5 s) using a wireless HR monitor (RCX5; Polar Electro, Kempele, Finland). RPE was evaluated using a 10-point scale (21) at the end of each trial.

#### 2.3.5. Data analyses

The anteroposterior and vertical GRF components were analyzed after they had been filtered at 40 Hz and 60 Hz, respectively, using a low-pass Butterworth filter. The contact phase, between the foot strike and toe-off, had a detection threshold of 50 N for the vertical GRF component. The braking and propulsive phases were between the foot strike and braking-to-propulsion transition, and between the braking-to-propulsion transition and toe-off, respectively. All GRF values were normalized according to body weight.

EMG signals were filtered at 20–450 Hz using band-pass Butterworth filters, then rectified and smoothed at 60 Hz using low-pass Butterworth filters. The muscle activity for each phase was calculated as the average EMG (aEMG) amplitude, using maximum voluntary contraction as the reference. EMG data were reported for the contact, braking, and propulsive phases. The GRF data and EMG signals during 10 steps have been averaged individually. In the GRF data, impulse and averaged force for each phase were calculated. The rectified time course EMG signals were aEMG for each phase.

#### 2.3.6. Statistical analyses

Data are presented as means ± standard deviations. A commercially available statistical software (SigmaStat 2.03; SPSS, Inc.) was utilized. For comparisons of each variable among the trials, one-way repeated-measures analysis of variance (repeated ANOVA) was used to compare the main effect. When ANOVA found significant main effect, *post hoc* test (Tukey method) was performed to identify specific pairwise differences. The level of statistical significance was set at *p* < 0.05.

## 3. Results

Table 1 shows walking speed variables.

### 3.1. GRFs comparison between SW, FW, and Run

**Figure 3:**
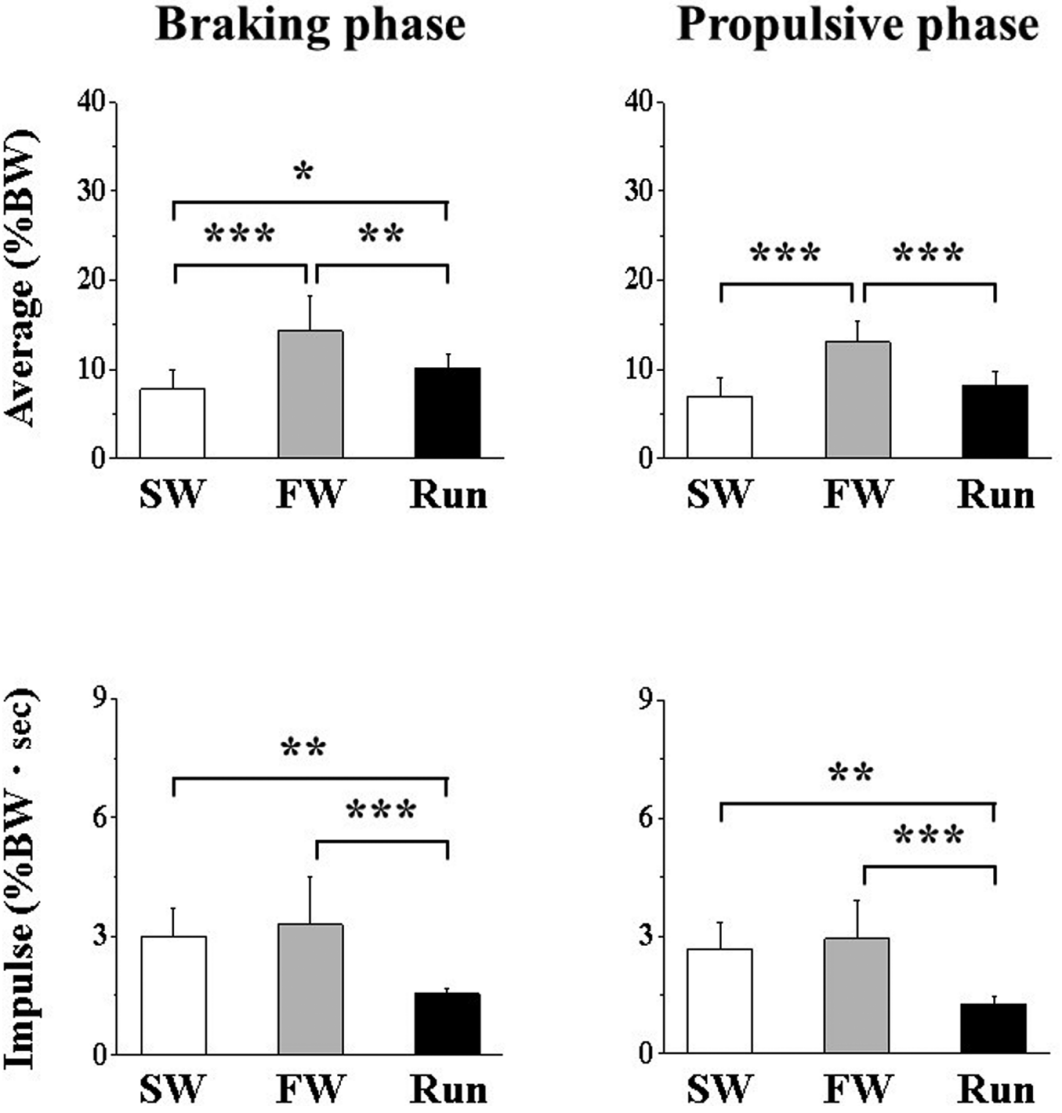
Anteroposterior component of ground reaction force during the braking (**A,B**) and propulsive (**C,D**) phases. Values are means ± SD. Significant difference between trials (*p < 0.05, **p < 0.01, ***p < 0.001). SW; Slow walk trial. FW; Fast walk trial. Run; Run trial.

Figure 3 shows the anteroposterior average and impulse GRFs. The impulse was significantly lower for Run than for SW or FW during the braking phase and during the propulsive phase. During the braking phase, the average force was significantly higher for FW and Run than for SW; it was higher for FW than for Run. During the propulsive phase, the average force was significantly higher for FW than for SW or Run.

**Figure 4:**
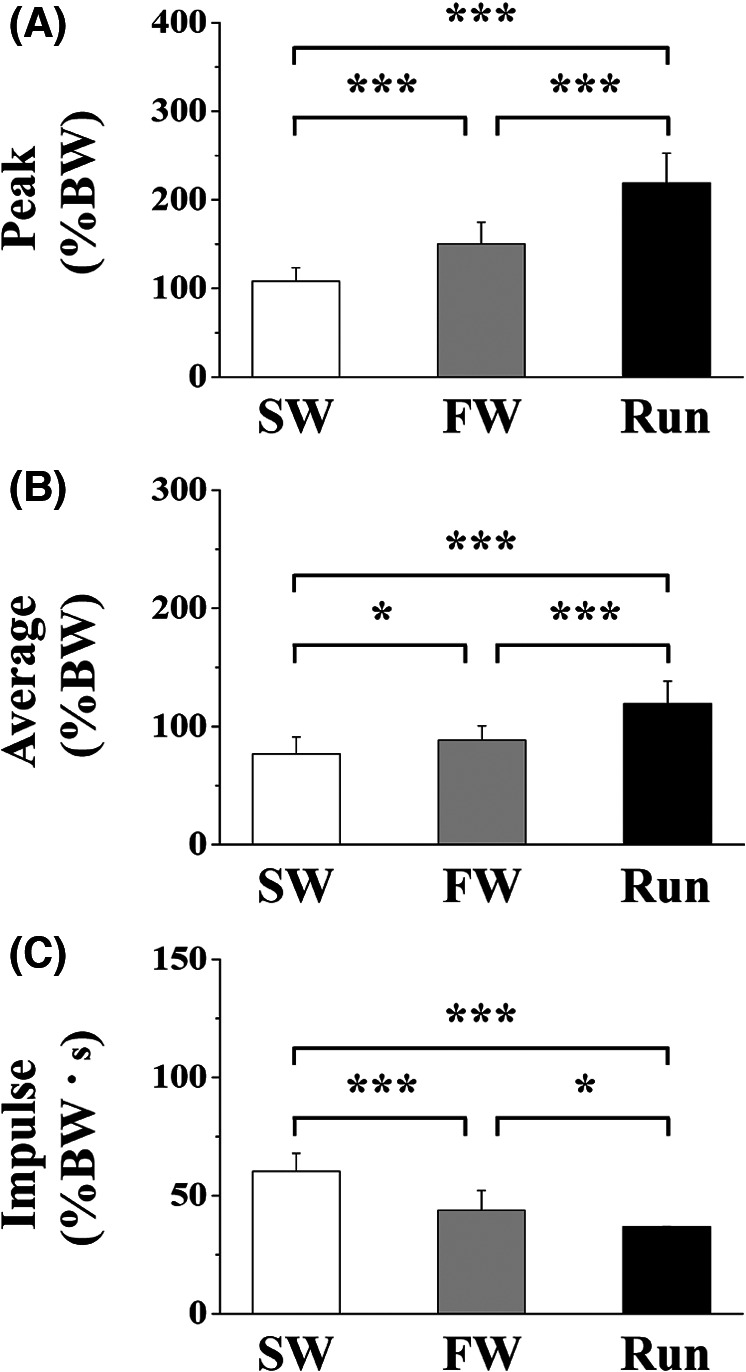
The peak (**A**), average (**B**) and impulse (**C**) of vertical component of ground reaction force. Values are means ± SD. Significant difference between trials (*p < 0.05, **p < 0.01, ***p < 0.001). SW; Slow walk trial. FW; Fast walk trial. Run; Run trial.

Figure 4 shows the vertical peak, average, and impulse GRFs. The peak force was significantly higher for FW and Run than for SW during the contact phase; it was significantly higher for Run than for FW. The average force was significantly higher for FW and Run than for SW during the contact phase; it was significantly greater for Run than for FW. The impulse was significantly lower for FW and Run than for SW during the contact phase; it was lower for Run than for FW.

### 3.2. EMGs comparison between SW, FW, and Run

**Figure 5:**
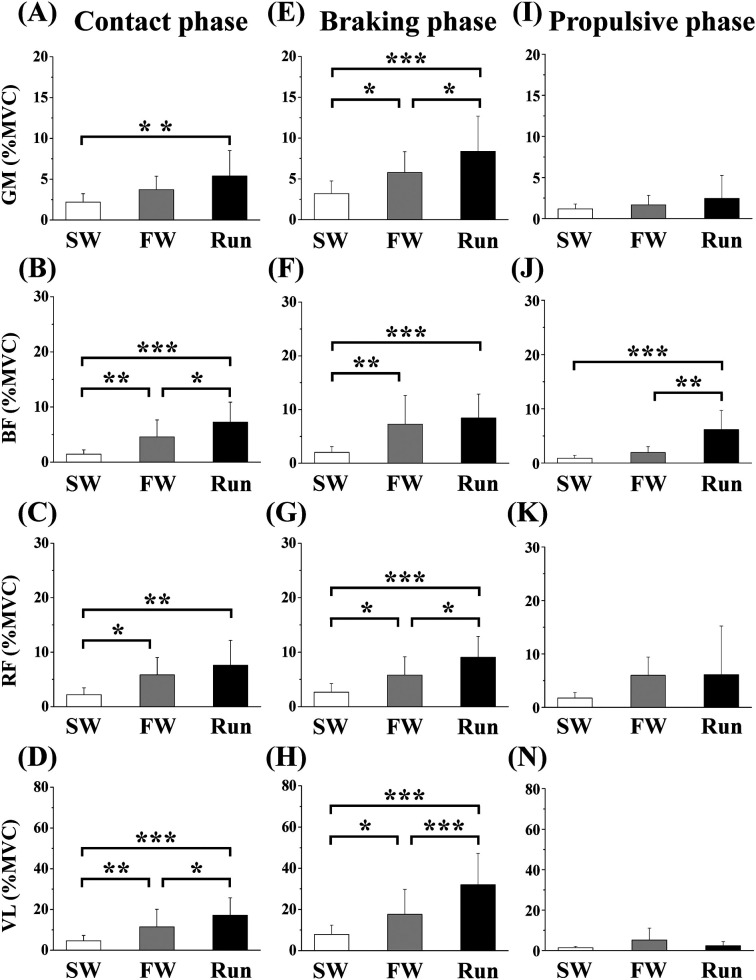
Averaged surface electromyography (aEMG) of femoral muscles during the contact (**A—D**), braking (**E—F**) and propulsive (**I—N**) phases. Values are means ± SD. Significant difference between trials (*p < 0.05, **p < 0.01, ***p < 0.001). SW; Slow walk trial. FW; Fast walk trial. Run; Run trial. GM; Gluteus maximus, BF; Biceps femoris, RF; Rectus femoris, VL; Vastus lateralis.

Figure 5 shows the aEMGs for the GM, BF, RF, and VL during the contact, braking, and propulsive phases. The contact phase aEMG for the GM was significantly higher in Run than in SW. The braking phase aEMG for the GM was significantly higher in Run than in SW and FW; it was higher in FW than in SW. The contact phase aEMG for the BF was significantly higher in Run than in SW or FW; it was higher in FW than in SW. The braking phase aEMG for the BF was significantly higher in FW and Run than in SW. Furthermore, the propulsive phase aEMG for the BF was higher in Run than in SW or FW. The contact phase aEMG for the RF was significantly higher in FW and Run than in SW. The braking phase aEMG for the RF was significantly higher in Run than in SW or FW; it was higher in FW than in SW. The contact phase aEMG for the VL was higher in Run than in SW or FW; it was higher in FW than in SW. The braking phase aEMG for the VL was higher in Run than in SW or FW; it was higher in FW than in SW.

**Figure 6:**
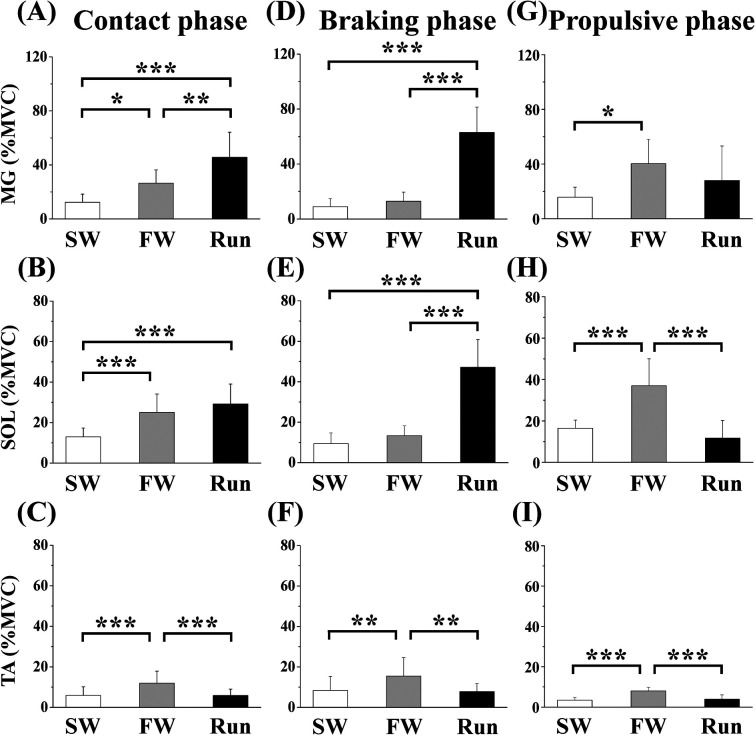
Averaged surface electromyography (aEMG) of lower leg muscles during the contact (**A—C**), braking (**D—F**) and propulsive (**G—I**) phases. Values are means ± SD. Significant difference between trials (*p < 0.05, **p < 0.01, ***p < 0.001). SW; Slow walk trial. FW; Fast walk trial. Run; Run trial. MG; Gastrocnemius medialis, SOL; Soleus, TA; Tibialis anterior.

Figure 6 shows the aEMGs for the MG, SOL, and TA during the contact, braking, and propulsive phases. The contact phase aEMG for the MG was significantly higher in Run than in SW or FW; it was higher in FW than in SW. The braking phase aEMG for the MG was significantly higher in Run than in FW or SW. The propulsive phase aEMG for the MG was significantly higher in FW than in SW. The contact phase aEMG for the SOL was significantly higher in Run and FW than in SW. The braking phase aEMG for the SOL was significantly higher in Run than in SW or FW. The propulsive phase aEMG for the SOL was significantly higher in FW than in SW or Run. The aEMG for the TA during all phases was significantly higher in FW than in SW or Run.

### 3.3. HR and RPE

Table 2 shows the HR and RPE. HR was significantly higher in FW and Run than in SW, but it did not significantly differ between FW and Run. RPE for breath (RPE_breath_) was significantly higher in FW and Run than in SW, with no difference between FW and Run. RPE for leg muscles (RPE_leg_) was significantly higher in FW and Run than in SW, and it was higher in FW than in Run.

**Table 2:** Hr and RPE.

		Slow walk	Fast walk	Run	Post hoc test (*p*-value)		
		(SW)	(FW)	(Run)	SW/FW	FW/Run	SW/Run
**HR (bpm)**	90 ± 14	147 ± 17	145 ± 19	<0.001	0.851	<0.001
**RPE**	**breath**	1.3 ± 0.5	3.5 ± 1.2	2.8 ± 0.7	<0.001	0.101	<0.01
**leg**	1.3 ± 0.5	4.9 ± 1.4	3.4 ± 1.1	<0.001	<0.01	<0.001

Values are means ± SD. HR; heart rate. RPE; rating of perceived exertion.

## 4. Discussion

The present study compared GRF and lower limb muscle activity between FW and running at equivalent speeds. Consequently, anteroposterior GRF was greater during FW than during Run, whereas vertical GRF was greater during Run than during FW. Moreover, muscle activity during the braking phase was lower in FW than in Run, while it was higher in FW during the propulsive phase. These findings suggest that FW causes less mechanical stress during impact and produces greater propulsive force, compared with running at an equivalent speed.

Despite equivalent speed, the impulse and average of anteroposterior GRF were significantly greater during FW than during running. In contrast, the average vertical GRF was higher during running than during FW. These results may be related to differences in locomotion (22). We (13) reported that EE and carbohydrate oxidation during walking were enhanced in a non-linear manner with increasing speed. Peak GM activity generally occurs immediately after foot-ground contact (23). In the present study, aEMGs for the GM, RF, and VL during braking phase were greater in Run than in FW. aEMGs during breaking phase showed similar trend for average vertical GRF. In the braking phase of running, the lower limb joints (i.e., hip, knee, and ankle joints) flexed because of the impact during ground contact. In the subsequent propulsive phase, these joints were extended, and the mechanical energy exchange involved elastic energy storage and release (24). Also, the peak of vertical GRF was significantly greater during running than during FW, and it is advantageous for the mechanical energy from exchange stretch- shortening cycle perspective (25).

Gazendam & Hof (23) reported minor hamstring differences in the walking and running profiles, but major differences in the EMG profiles were observed for the lower limb muscles. In the present study, aEMGs for the MG and SOL during braking phase were higher in running than in walking. However, aEMG for the MG during the propulsive phase was significantly higher in FW than in SW, and aEMG for the SOL was significantly higher in FW than in SW and running. Less knee flexion with greater ankle plantarflexion decreased mechanical efficiency at fast speed (26), and it may augment mechanical work, thus decreasing mechanical efficiency (27). Furthermore, impulse and average anteroposterior GRFs were significantly higher in FW than in running during the propulsive phase. Therefore, increased muscle activities during push-off might be involved in the greater EE during walking (28). The present study also demonstrated differences in aEMGs for the TA during FW and Run. The EMG of TA was significantly higher in FW than in SW and Run during all phases. The increased aEMG for the TA during FW would contribute to maintaining the ankle joint in a dorsiflexed position, thus improving ankle joint stability while walking (29). Furthermore, the augmented muscle activities of lower limb muscles might explain significantly higher score of RPE_leg_ in FW than in Run.

As limitations of the present study, joint torques and powers were not evaluated. Also, the evaluations of GRFs were limited during 4 min of exercise (walking or running). In further study, determination of GRFs during actual interval FW exercise would be valuable.

## 5. Conclusion

FW resulted in a smaller impact (i.e., vertical GRF) than running at an equivalent speed. During the contact phase, the average muscle activities of GM, RF, and SOL were not significantly different between FW and running. However, further analyses presented that the activity patterns of lower limb muscles differed between FW and running, even at equivalent speeds. In running, GM, RF, VL, MG, and SOL were mainly activated during the braking phase than the propulsive phase. On the other hand, in FW, the muscle activity of SOL during the propulsive phase was increased than during the braking phase. Therefore, FW causes smaller mechanical stress than running at an equivalent speed, but it highly activates lower limb muscles. These notions may be useful for designing exercise program for health promotion, particularly in individuals with orthopedic or other medical issues (e. g., joint injuries, type 2 diabetes, and obesity).

## Data Availability

The original contributions presented in the study are included in the article/Supplementary Material, further inquiries can be directed to the corresponding authors.
